# Supplementary material for: Bronchoalveolar Lavage Fluid from COPD Patients Reveals More Compounds Associated with Disease than Matched Plasma
Source: Metabolites. 2019 Jul 25;9(8):157. doi: 10.3390/metabo9080157 (PMC6724137; doi:10.3390/metabo9080157)
Supplement: Supplementary file 1 [file metabolites-09-00157-s001.pdf]

Supplemental

Table S1. SPIROMICs Cohort Characteristics.

	Never Smokers	Smoking Controls	COPD	p-value
n	196	929	1842	0
Sex, % men	38	48	57	5.28E-10
Race, % (white,black,asian,other)	69,22,2,6	68,27,1,5	81,15,1,2	5.00E-04
Current smokers, %	0	51	35	1.54E-54
Age, yr	56 (50-64)	61 (52-68)	66 (59-71)	2.82E-67
Pack-years	0 (0-0)	37 (29-50)	46 (35-62)	1.49E-118
Body mass index	28.52 (4.96)	28.98 (5.07)	27.35 (5.31)	1.02E-11
Bronchitis Attack, %	22 (42)	39 (49)	52 (50)	5.00E-04
Chronic bronchitis, %	2 (14)	17 (38)	24 (43)	7.37E-18
Exacerbations/yr	0.04 (0.19)	0.22 (0.66)	0.55 (1.03)	5.00E-04
Emphysema, %	0.07 (0.03-0.23)	0.11 (0.04-0.39)	4.33 (0.82-14.19)	1.20E-98
FEV1 %	101.78 (11.68)	97.16 (13.38)	60.98 (22.96)	0
FEV1/FVC	81 (77-84)	77 (73-81)	54 (41-63)	0

Data are presented as median (interquartile range) or mean ± SD.; Emphysema, %: % Emphysema voxels (< -950 Hounsfield units) in lung CT image; Bronchitis attack: At least one episode of bronchitis.; Exacerbations/yr: # of exacerbations in last year. ;Chronic bronchitis: Daily productive cough for at least 3 months in the previous 2 consecutive years.; % FEV1: Postbronchodilator % predicted forced expiratory volume in one Second; FEV1/FVC: Ratio of forced expiratory volume in one second to forced vital capacity.

Table S2. Regression Design.

Phenotype	Regression	Covariates
% Emph	beta	Age, Sex,% FEV1, Smoker, Site, Race
FEV1:% predicted	linear	
FEV1/FVC	beta	Age, Sex, Asthma, Site, Race
Chronic Bronchitis		Age, Sex, Smoker, Site, Race, BMI, % FEV1
Exacerbations	Negative binomial	Sex, Race, % FEV1
Sex	linear	Site
Age	linear	Site
Smoker	linear	Site
Menopause	linear	Site
cntNeut	linear	Site
cntLymph	linear	Site
cntEos	linear	Site
Hct	linear	Site
Hgb	linear	Site
BAL cntNeut	linear	Site
BAL cntLymph	linear	Site
BAL cntEos	linear	Site
BAL cntMonocyte	linear	Site
BAL cntMacrophage	linear	Site

Regressions listed in table were used for compounds with <20% missingness. Tobit regression was performed for all compounds with >20% missingness for all compounds Tobit regression covariates were the same as those listed above with the addition of batch.; % Emph:%; Emphysema voxels (< -950 Hounsfield units) in lung CT image ;FEV1 % predicted: Postbronchodilator % predicted forced expiratory volume in one Second; FEV1/FVC: Ratio of forced expiratory volume in one second to forced vital capacity.; Exacerbations: # of exacerbations in last year; Smoker: Current Smoker; BMI: Body mass index; cntNeut: Neutrophil Count in Plasma; cntLymph: Lymphocyte Count in Plasma; cntEos: Eosinophil Count in Plasma; Hct: % hematocrit from coulter counter (complete blood count).; Hgb: Hemoglobin from coulter counter (complete blood count); BAL cntNeut: Neutrophil Count in BAL; BAL cntLymph: Lymphocyte Count in BAL ;BAL cntEos: Eosinophil Count in BAL; BAL cntMonocyte: Monocyte Count in BAL ;BAL cntMacrophage: Macrophage Count in BAL

Table S3. Compound Types Searched.

<table>
<tr><th>Compound Type</th><th>Regular Expression</th></tr>
<tr><td>phospholipids'</td><td>'^p[ascie]\\('</td></tr>
<tr><td>Phosphatidate</td><td>^pa\\('</td></tr>
<tr><td>phosphocholine</td><td>'^pc\\('</td></tr>
<tr><td>phosphoethanolamine</td><td>'^pe\\('</td></tr>
<tr><td>phosphatydlserine</td><td>'^ps\\('</td></tr>
<tr><td>phosphoinositol</td><td>'^pi\\('</td></tr>
<tr><td>lysopcs</td><td>'lysopc'</td></tr>
<tr><td>lysopes</td><td>‘lysope’</td></tr>
<tr><td>lysops</td><td>'lysop[ce]'</td></tr>
<tr><td>carnitine</td><td>'carn'</td></tr>
<tr><td>Amino acid</td><td>ala |ala$|alanine|arg |arg$|arginine|asn |asn$|asparagine|asp |asp$|aspartic|cys |cys$|cysteine|gln |gln$|glutamine|glu |glu$|glutamic|glutamat|gly |gly$| glycine|his |his$|histidine|ile |ile$|isoleucine|leu |leu$|leucine|lys |lys$|lysine|met |met$|methionine|phe |phe$|phenylalanine|pro |pro$|proline|^pyl | pyl$|pyrrolysine|ser |ser$|serine|sec |sec$|selenocysteine|thr |thr$|threonine|trp|tryptophan|tyr |tyr$|tyrosine|val |val$|valine</td></tr>
<tr><td>Fatty acid</td><td>'ic acid'</td></tr>
<tr><td>Alanine</td><td>"ala |ala$|alanine",</td></tr>
<tr><td>Arginine</td><td>"arg |arg$|arginine",</td></tr>
<tr><td>Asparagine</td><td>"asn |asn$|asparagine",</td></tr>
<tr><td>Aspartic acid</td><td>"asp |asp$|aspartic",</td></tr>
<tr><td>Cysteine</td><td>"cys |cys$|cysteine",</td></tr>
<tr><td>Glutamine</td><td>"gln |gln$|glutamine",</td></tr>
<tr><td>Glutamic acid</td><td>"glu |glu$|glutamic|glutamat",</td></tr>
<tr><td>Glycine</td><td>"gly |gly$| glycine",</td></tr>
<tr><td>Histidine</td><td>"his |his$|histidine",</td></tr>
<tr><td>Isoleucine</td><td>"ile |ile$|isoleucine",</td></tr>
<tr><td>Leucine</td><td>"leu |leu$|leucine",</td></tr>
<tr><td>Lysine</td><td>"lys |lys$|lysine",</td></tr>
<tr><td>Methionine</td><td>"met |met$|methionine",</td></tr>
<tr><td>Phenylalanine</td><td>"phe |phe$|phenylalanine",</td></tr>
<tr><td>Proline</td><td>"pro |pro$|proline",</td></tr>
<tr><td>Pyrrolysine</td><td>"^pyl | pyl$|pyrrolysine",</td></tr>
<tr><td>Serine</td><td>"ser |ser$|serine",</td></tr>
<tr><td>Selenocysteine</td><td>"sec |sec$|selenocysteine",</td></tr>
<tr><td>Threonine</td><td>"thr |thr$|threonine",</td></tr>
<tr><td>Tryptophan</td><td>"trp|tryptophan",</td></tr>
<tr><td>Tyrosine</td><td>"tyr |tyr$|tyrosine",</td></tr>
<tr><td>Valine</td><td>"val |val$|valine"</td></tr>
</table>

Table S4. Institutional Review Board Approval Documentation for SPIROMICS.

Participating Center	Institution Title for Review Board	Protocol Number
**SPIROMICS**		
Columbia University Medical Center	Columbia University Medical Center IRB	IRB-AAAE9315
Johns Hopkins University	Johns Hopkins Medicine Institutional Review Boards (JHM IRB)	NA_00035701 / CIR00004922
National Jewish Health	National Jewish IRB	HS-2678
Temple University	Temple University Office for Human Subjects Protections Institutional Review Board	21416
University of Alabama at Birmingham	The University of Alabama at Birmingham Institutional Review Board for Human Use	F120906004
University of California, Los Angeles	UCLA Office of the Human Research Protection Program	10-001740-CR-00004
University of California, San Francisco	UCSF Human Research Protection Program, Committee on Human Research	10-03169
University of Illinois at Chicago	UIC Office for the Protection of Research Subjects (OPRS)	2013-0939
University of Iowa	The University of Iowa Human Subjects Office/Institutional Review Board (IRB)	201308719
University of Michigan	Medical School Institutional Review Board (IRBMED)	HUM00036346
University of North Carolina at Chapel Hill	UNC-CH Office of Human Research Ethics (OHRE) Non-Biomedical IRB	10-0048
University of Utah	The University of Utah Institutional Review Board	00027298
Wake Forest University	Wake Forest University Health Sciences Office of Research Institutional Review Board	IRB00012805

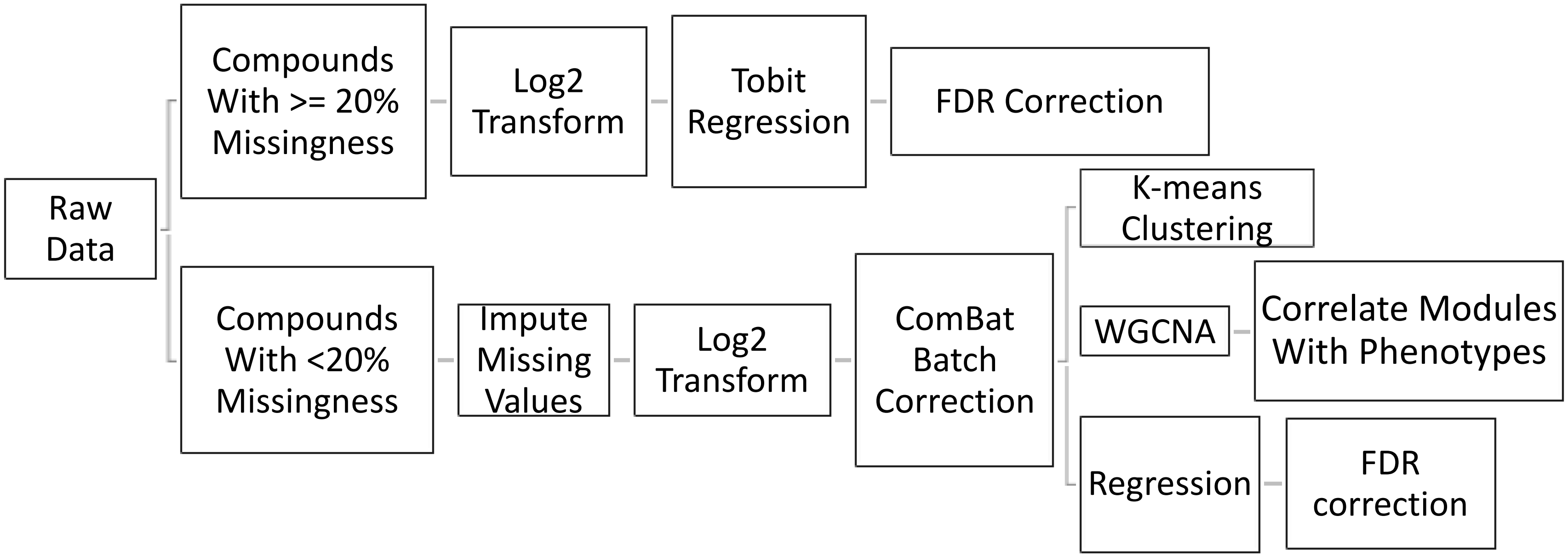

Figure S1. Analysis Procedure.

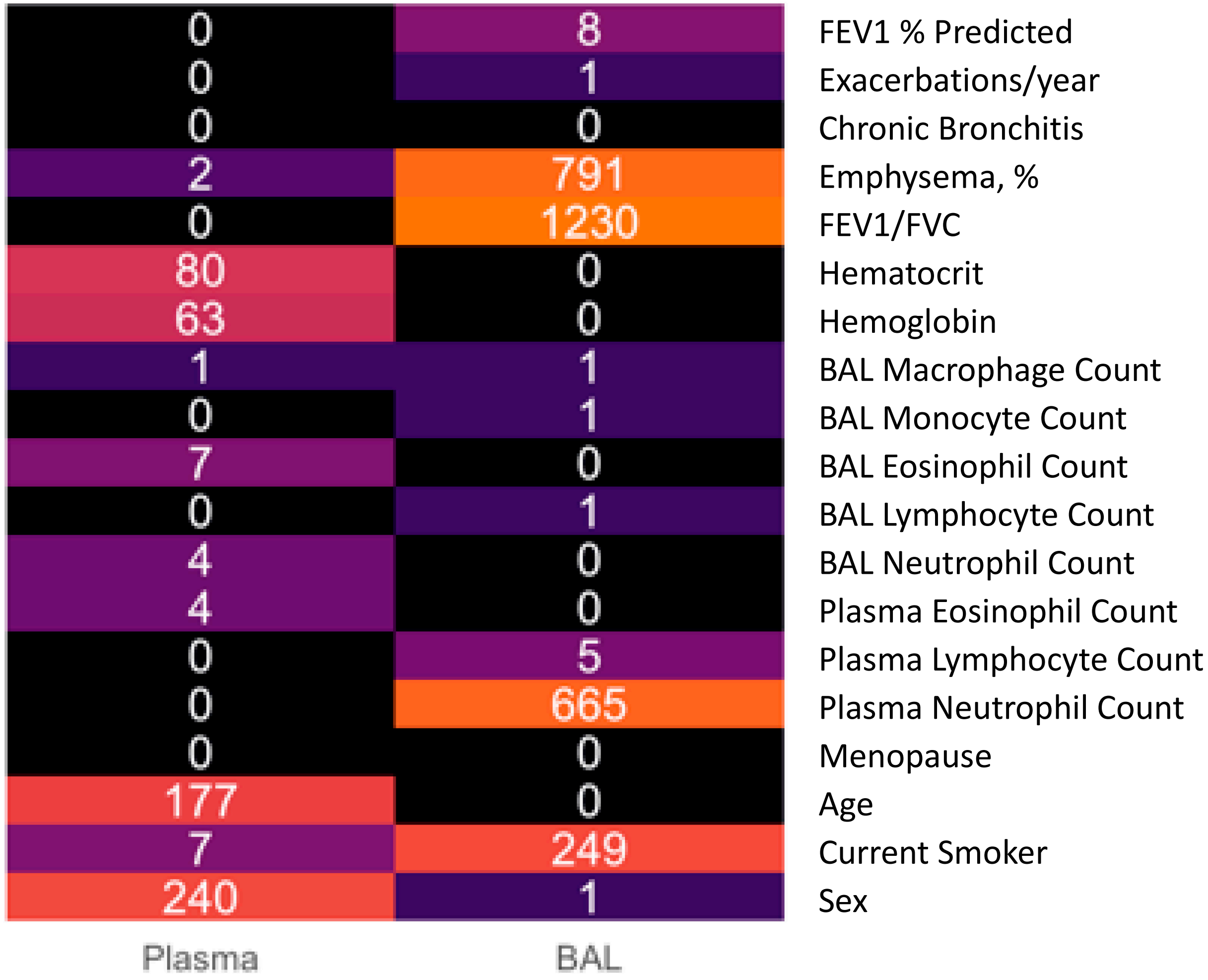

Figure S2. Heatmap of Associations Between Compounds and Phenotypes.

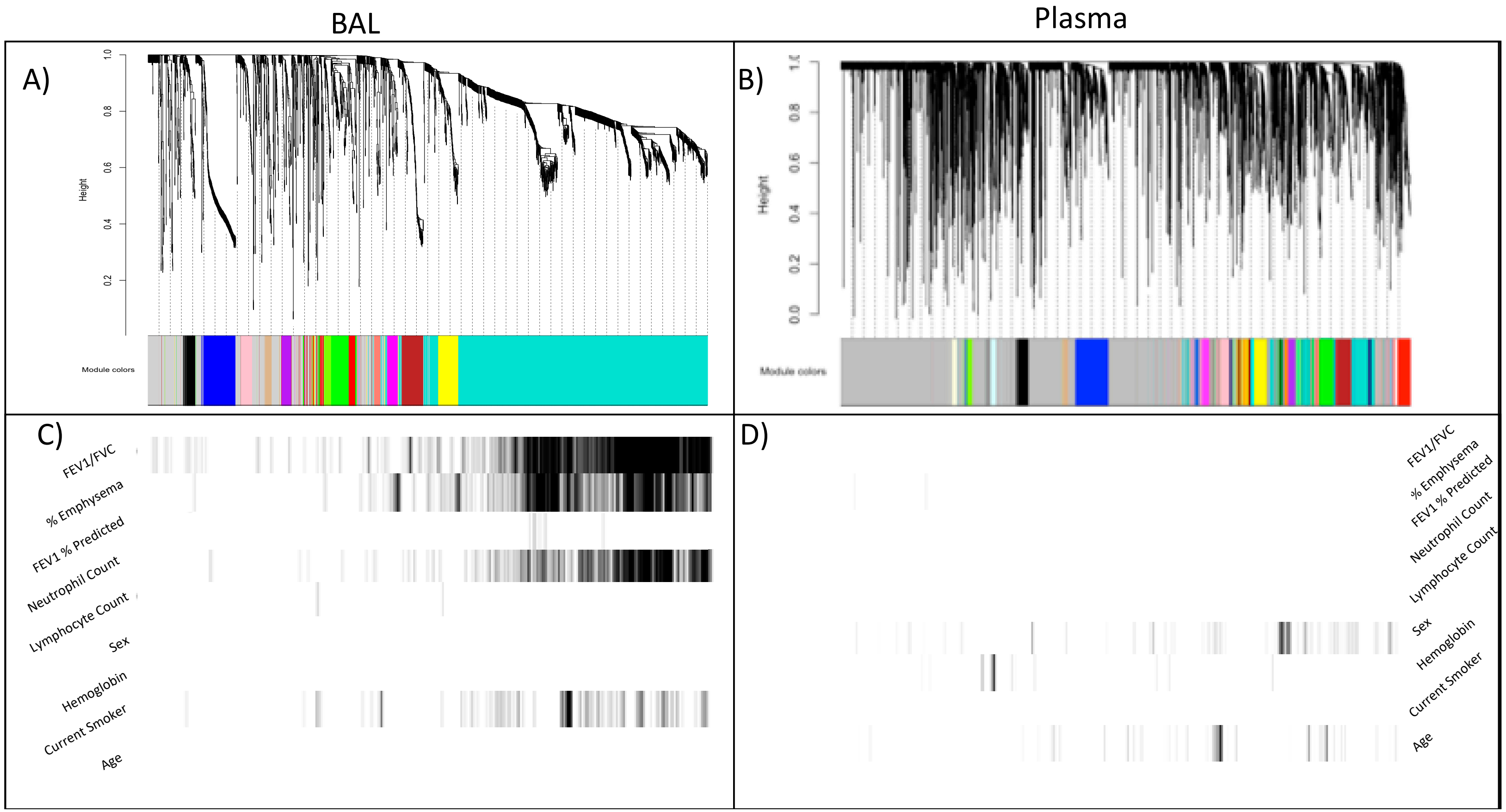

Figure S3. Significant Compounds in Correspondence with WGCNA Modules (**A**)WGCNA Derived BAL compound modules, colors correspond to modules compiled by WGCNA, dendrograms were created using average linkage hierarchical clustering on a dissimilarity matrix within the WGCNA software package **B**) WGCNA Derived plasma compound modules. **C**) BAL compounds individually associated with selected COPD sub-phenotypes, cell counts, and clinical variables. Order matches position of compounds in WGCNA module in A. **D**) Plasma compounds individually associated with selected COPD sub-phenotypes, cell counts, and clinical variables. Order matches position of compounds in WGCNA module in B.

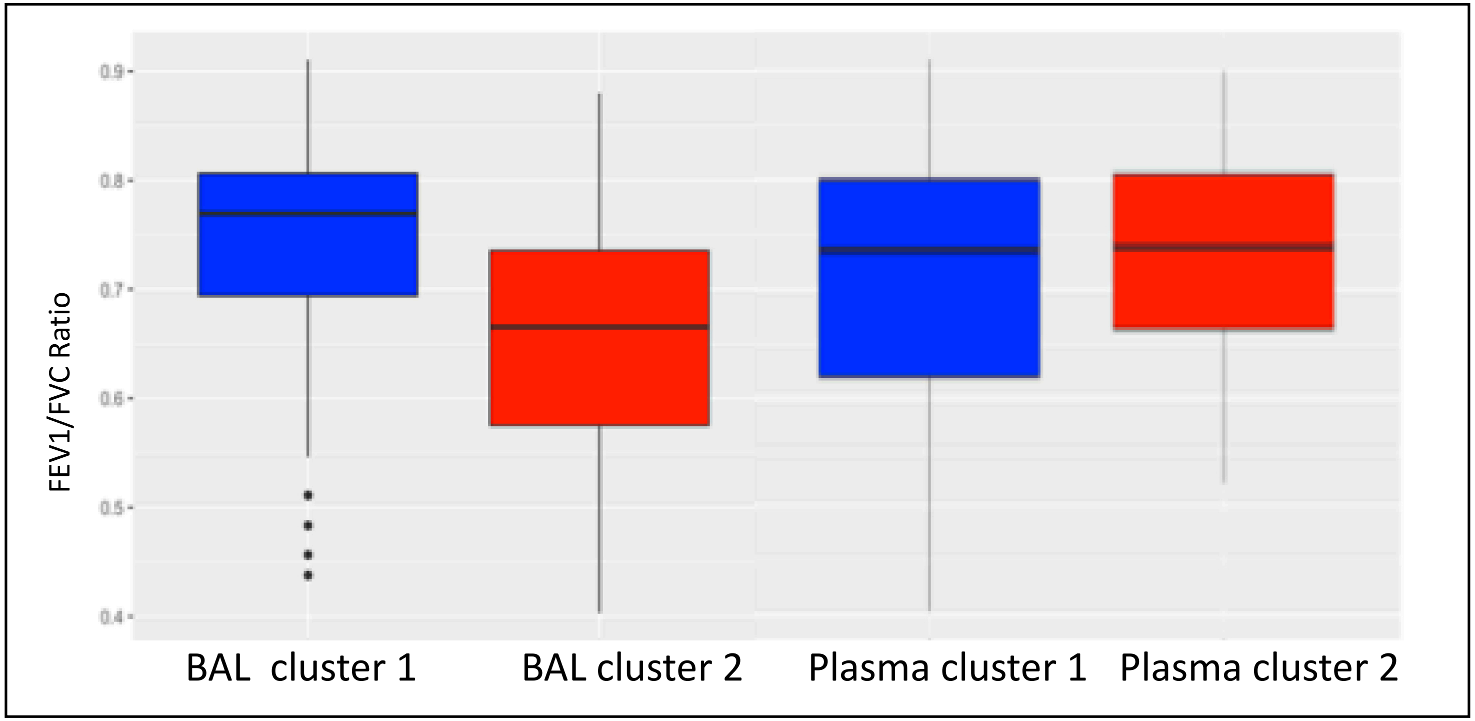

Figure S4. K-means Clustering of Subjects Based on Compound Profiles.

K-means clustering of subjects based upon compound profiles, demonstrating distribution of FEV1/FVC within each of the two clusters for both BAL and plasma.

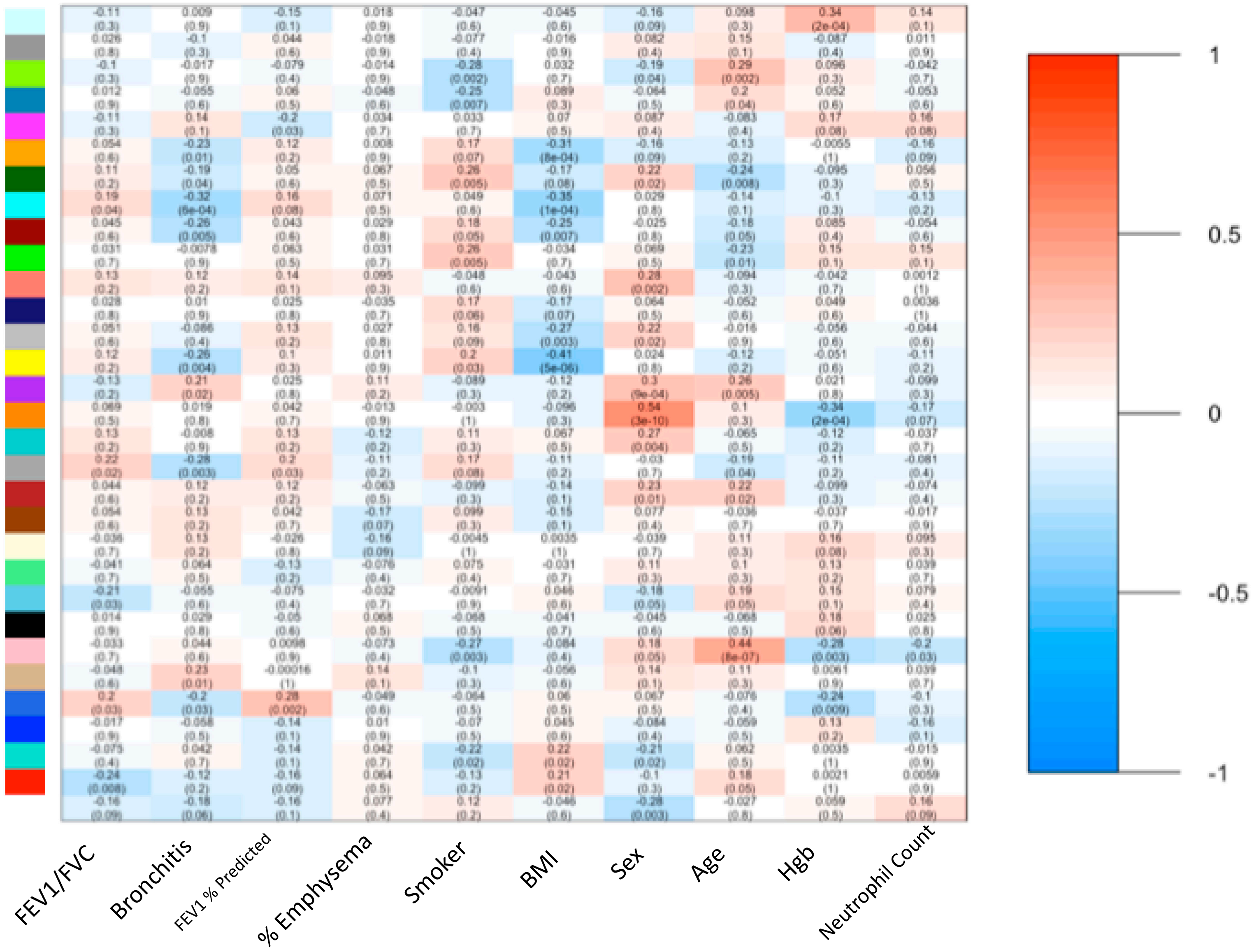

Figure S5. Full WGCNA Module Eigenvalue to Phenotype. Relationship in Plasma.

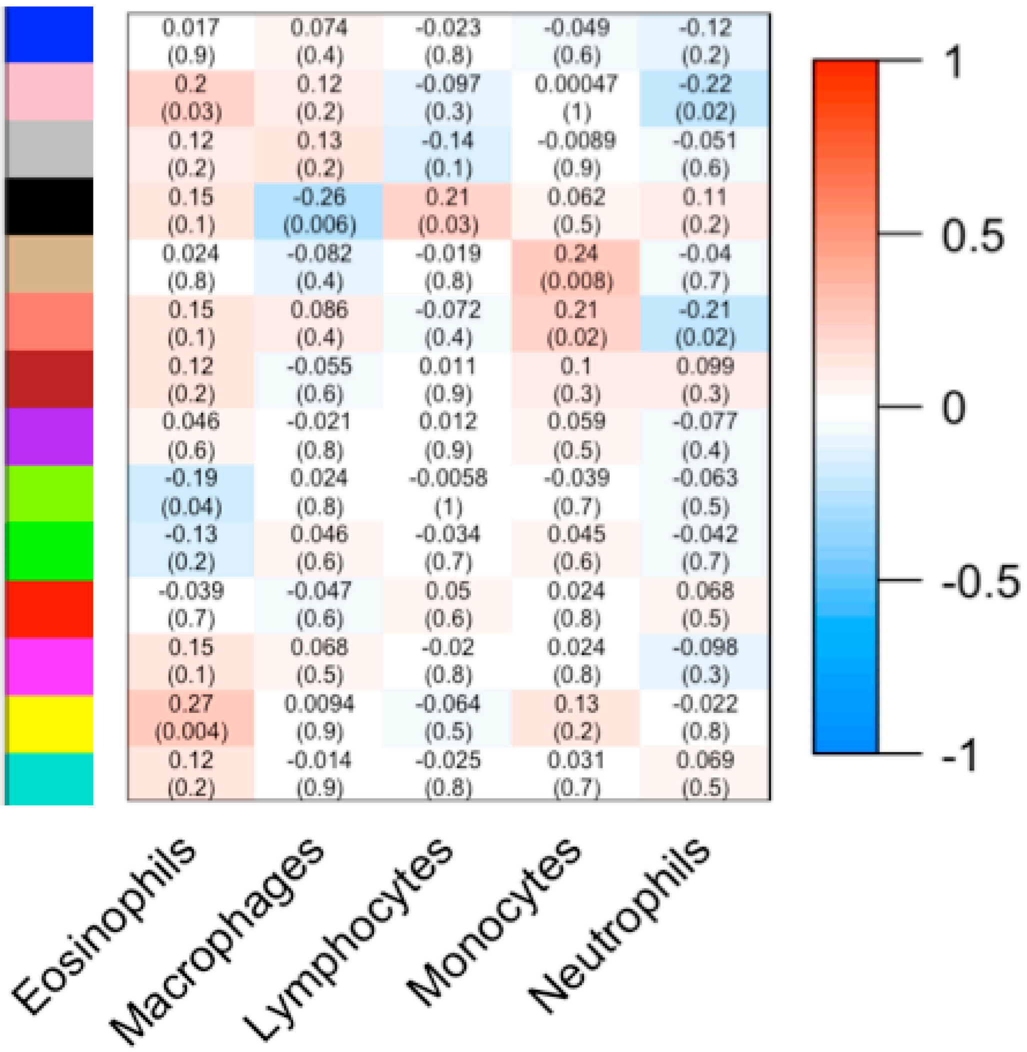

Figure S6. WGCNA Module Eigenvalue to Phenotype Relationship in. BAL for BAL Cell Counts.
